# Effect of Y_6_, an epigallocatechin gallate derivative, on reversing doxorubicin drug resistance in human hepatocellular carcinoma cells

**DOI:** 10.18632/oncotarget.15964

**Published:** 2017-03-07

**Authors:** Yan Wen, Rui-Qiang Zhao, Yun-Kai Zhang, Pranav Gupta, Li-Xiang Fu, An-Zhou Tang, Bu-Ming Liu, Zhe-Sheng Chen, Dong-Hua Yang, Gang Liang

**Affiliations:** ^1^ Department of Pharmacy, The First Affiliated Hospital of Guangxi Medical University, Nanning 530021, P.R. China; ^2^ Department of Pharmaceutical Sciences, College of Pharmacy and Health Sciences, St. John's University, Queens, NY 11439, USA; ^3^ Department of Biochemistry and Molecular Biology, School of Preclinical Medicine, Guangxi Medical University, Nanning 530021, P.R. China; ^4^ College of Pharmacy, Guangxi Medical University, Nanning 530021, P.R. China; ^5^ Department of Otolaryngology, The First Affiliated Hospital of Guangxi Medical University, Nanning 530021, P.R. China; ^6^ Guangxi Key Laboratory of Traditional Chinese Medicine Quality Standards, Guangxi Institute of Chinese Medicine and Pharmaceutical Sciences, Nanning 530022, P.R. China

**Keywords:** Y_6_, epigallocatechin-3-gallate (EGCG), multidrug resistance, resistance reversal agent, doxorubicin

## Abstract

Cancer cells can acquire resistance to a wide variety of diverse and unrelated drugs, this phenomenon is termed multidrug resistance (MDR). Multidrug resistance has been an obstacle to the success of cancer chemotherapy. The present study investigated the reversal effect of Y_6_, a new compound obtained by chemically modifying the structure of epigallocatechin-3-gallate (EGCG) extracted from green tea. Y_6_ was proven to be effective in inhibiting cell proliferation and reversing drug resistance in doxorubicin (DOX) resistant human hepatocellular carcinoma cells (BEL-7404/DOX). BEL-7404/DOX cells were treated with either doxorubicin combination regimen (doxorubicin plus Y_6_ or epigallocatechin-3-gallate or verapamil separately) or doxorubicin alone. The results showed that cell proliferation was inhibited and late cell apoptosis increased in the combination treatment group, especially in the group treated with doxorubicin plus Y_6_. Further analysis revealed that the expressions of hypoxia-inducible factor-1α and multidrug resistance 1/P-glycoprotein decreased at both messenger RNA and protein levels by treatments with combined drugs compared to doxorubicin alone. Our results indicated that Y_6_, as a drug resistance reversal agent, increased the sensitivity of drug resistant cells to doxorubicin. The mechanisms of actions of Y_6_ in reversal effect were associated with the decreased expression of hypoxia-inducible factor-1α and multidrug resistance 1/P-glycoprotein.

## INTRODUCTION

Liver cancer is a malignancy of high incidence and mortality. It is estimated that nearly 750,000 new cases and more than 690,000 cancer deaths occur annually worldwide. Unfortunately, half of these cases were estimated to occur in China [1]. Among the liver cancers, hepatocellular carcinoma (HCC) is the major histological subtype. Various anticancer drugs such as tamoxifen, octreotide, interferon, and interleukin-2 are used in the treatment of HCC; however, these drugs are not specific for HCC and demonstrate low treatment efficacy [2]. Multidrug resistance (MDR) to anticancer drugs is the most common cause of failure in cancer chemotherapy [3].

The mechanisms of resistance to anticancer drugs consist of changes of pharmacokinetic or tumor micro-environment, cancer cell-specific factors which include increased drug efflux or decreased drug influx, drug inactivation, drug target modification, and apoptosis evasion [4]. Of these mechanisms, the increased drug efflux/reduced drug accumulation in the tumor cell appears to be a very common mechanism of MDR. Hydrophobic drugs are expelled from cells by activation of energy-dependent transport systems [5, 6]. The first of these transporters to be identified was P-glycoprotein (P-gp), the product of the human *MDR1* gene [7]. P-gp is a member of the large adenosine triphosphate (ATP)-binding cassette (ABC) family of proteins, also known as ABCB1. P-gp/ABCB1 has a molecular weight of 170 kDa and comprises of two nucleotide-binding domains (NBD1 and NBD2) and two transmembrane-binding domains (TMD1 and TMD2) [8, 9]. P-gp/ABCB1 utilizes energy from the hydrolysis of ATP to efflux drugs from intracellular to extracellular matrix, leading to decreased intracellular drug concentration [10]. Overexpression of P-gp/ABCB1 can produce MDR in cancer cells [11].

Over the past few decades, efforts were made to look for new compounds as resistance reversal agents to overcome MDR in tumor cells. Verapamil was one of the first generation of these MDR reversal agents [12]. But the effective concentration of verapamil in reversing MDR *in vitro* was too high to be achieved *in vivo* [13]. The dose of verapamil required was much larger than the clinically safe dose, resulting in toxic reactions in almost all patients. Although second and third generations of reversal agents were explored, they were inhibited respectively by P450 3A4 enzymes and by anticancer drugs [14–16]. In order to obtain more effective and safer resistance reversal agents, some natural products and their derivatives have been considered for use in combination with anticancer drugs [17].

**Figure 1:**
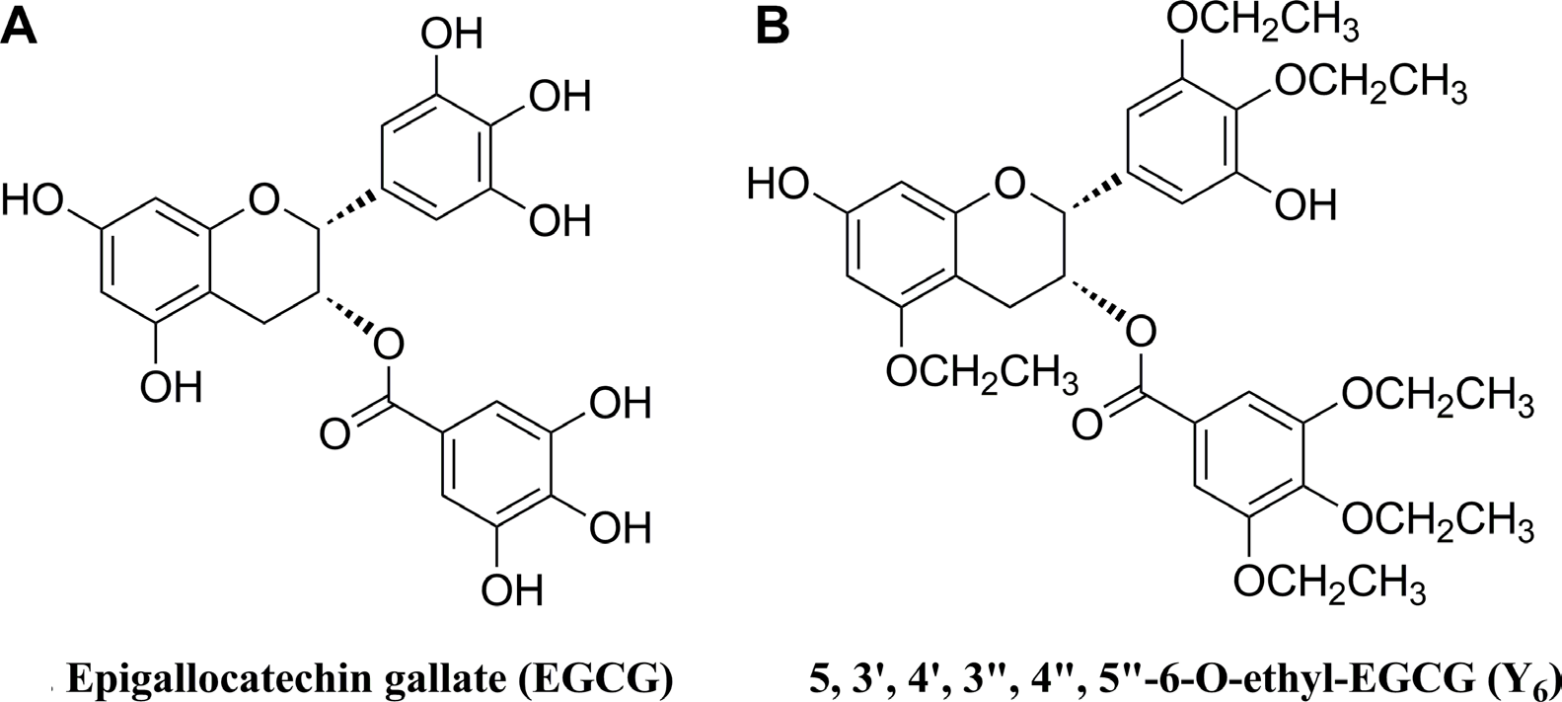
Chemical structures of two monomers of catechin

Epigallocatechin-3-gallate (EGCG) is one of the MDR reversal modulators (Figure 1A) [18]. It is the most abundant catechin in green tea polyphenols. A previous study revealed that EGCG could significantly inhibit the proliferation of human HCC cell BEL-7404/DOX *in vitro* and the tumor growth of the xenograft mouse model when it was administrated at lower doses with doxorubicin, compared to treatment with doxorubicin alone [19]. However, many phenolic hydroxyl groups are included in the structure of EGCG, which makes the compound unstable due to rapid oxidation, low lipid solubility, low bioavailability, and short duration of action. Therefore, its application became limited [20]. Y_6_ is an ethylation product of EGCG with strong stability (Figure 1B). The above limitations were overcome in Y_6_ because most of the phenolic hydroxyl groups were replaced by ethyl groups. In the present study, we evaluate the potential effect of Y_6_ as a reversal agent that specifically reverses ABC transporter-mediated MDR *in vitro*.

## RESULTS

### Y_6_ improved the chemosensitivity of BEL-7404/DOX cells to doxorubicin treatment

**Figure 2:**
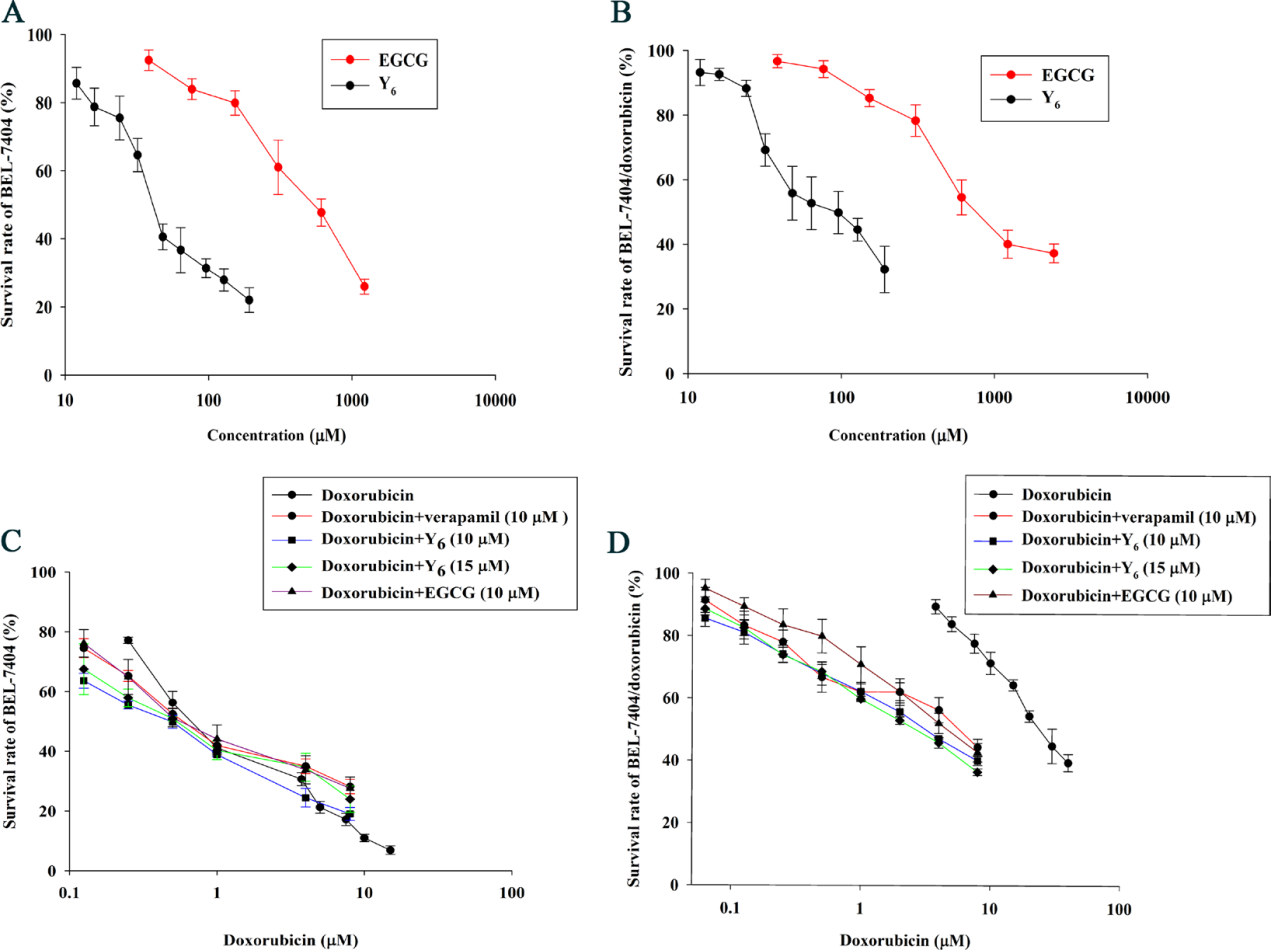
The Reversal effect of Y6, EGCG, and verapamil in parental and resistant cells (**A**) The cytotoxicity of EGCG and Y_6_ in the parental cell line BEL-7404. (**B**) The cytotoxicity of EGCG and Y_6_ in the doxorubicin-resistance cell line BEL-7404/DOX. (**C**) (**D**) the concentration-response curves of hepatocellular carcinoma cell lines (C) BEL-7404 and (D) BEL-7404/DOX treated with doxorubicin alone and doxorubicin combined with verapamil, EGCG, or Y_6_. Cell survival rate was determined by MTT assay. Points with error bars represent the mean ± SD. Experiments were performed for three independent times.

HCC cell lines (parental BEL-7404 and doxorubicin-selected resistant BEL-7404/DOX cells) were used to examine the cytotoxicity of doxorubicin, EGCG, and Y_6_ on cell proliferation using the 3-(4, 5-dimethylthiazol-2-y1)-2, 5-diphenyltetrazolium bromide (MTT) assay. As shown in Figure 2, higher concentration of doxorubicin is required to inhibit the same ratio of BEL-7404/DOX cell proliferation than that required in BEL-7404 cells. The IC_50_ value (44.14 μM) for doxorubicin in the BEL-7404/DOX cells was 29.2 times higher than that (1.51 μM) in the parental BEL-7404 cells (Table 1). The IC_50_ values of Y_6_ and EGCG in this pair of cell groups were > 50 μM. The concentrations for combined use with doxorubicin were non-cytotoxic and below the IC_10_ values (Figure 2).

**Table 1:** Reversal effect of Y_6_, EGCG, and VER in BEL-7404 cells and BEL-7404/DOX cells

Compounds	IC_50_ ± SD (μM)^a^ (Resistance folds)^b^	
	BEL-7404	BEL-7404/DOX
DOX	1.51 ± 0.14 (1.0)	44.14 ± 3.97 (29.2)
+VER 10 μM	1.47 ± 0.13 (1.0)	8.39 ± 1.43 (5.6)*
+EGCG 10 μM	1.49 ± 0.32 (1.0)	8.04 ± 1.08 (5.3)*
+Y_6_ 10 μM	1.29 ± 0.21 (0.9)	5.74 ± 0.73 (3.8)^*#^
+Y_6_ 15 μM	1.15 ± 0.31 (0.8)	4.36 ± 0.15 (2.9)^*#^

Cell survival was determined by MTT assay. a: Values represent means ± SDs of at least three independent experiments performed in triplicate. b: The fold reversal of MDR (values given in parentheses) was calculated by dividing the IC_50_ values of substrate in BEL-7404/DOX cells in the presence or absence of an inhibitor, or BEL-7404 cells with inhibitors, by the IC_50_ of BEL-7404cells without an inhibitor. *p < 0.05, significantly different from values obtained in the absence of an inhibitor; ^#^*p* < 0.05, significantly different from values obtained in the presence of inhibitors VER or EGCG.

The non-toxic doses of verapamil, EGCG, and Y_6_ were individually combined with a series of doxorubicin concentrations, and were used to treat BEL-7404 cells and BEL-7404/DOX cells. Among them, verapamil, a known chemosensitizer, was used as the positive control inhibitor of P-gp in this experiment. The MTT assay results showed that the IC_50_ values for doxorubicin was significantly reduced from 44.14 to 8.39 μM by verapamil, to 8.04 μM by EGCG, and to 5.74 μM and 4.36 μM by Y_6_ (10 μM) and Y_6_ (15 μM) respectively in the BEL-7404/DOX cells compared with doxorubicin alone, representing drug resistance folds decreasing from 29.2 to 5.6, 5.3, 3.8, and 2.9 respectively (Table 1). Combination groups were compared with the doxorubicin alone group, and the differences were statistically significant (*p < 0.05). These results indicated that the capability of Y_6_ in reversing drug resistance was higher than that of EGCG combined with doxorubicin (^#^*p* < 0.05) (Table 1, Figure 2).

### Y_6_ induced apoptosis in BEL-7404/DOX cells

**Figure 3:**
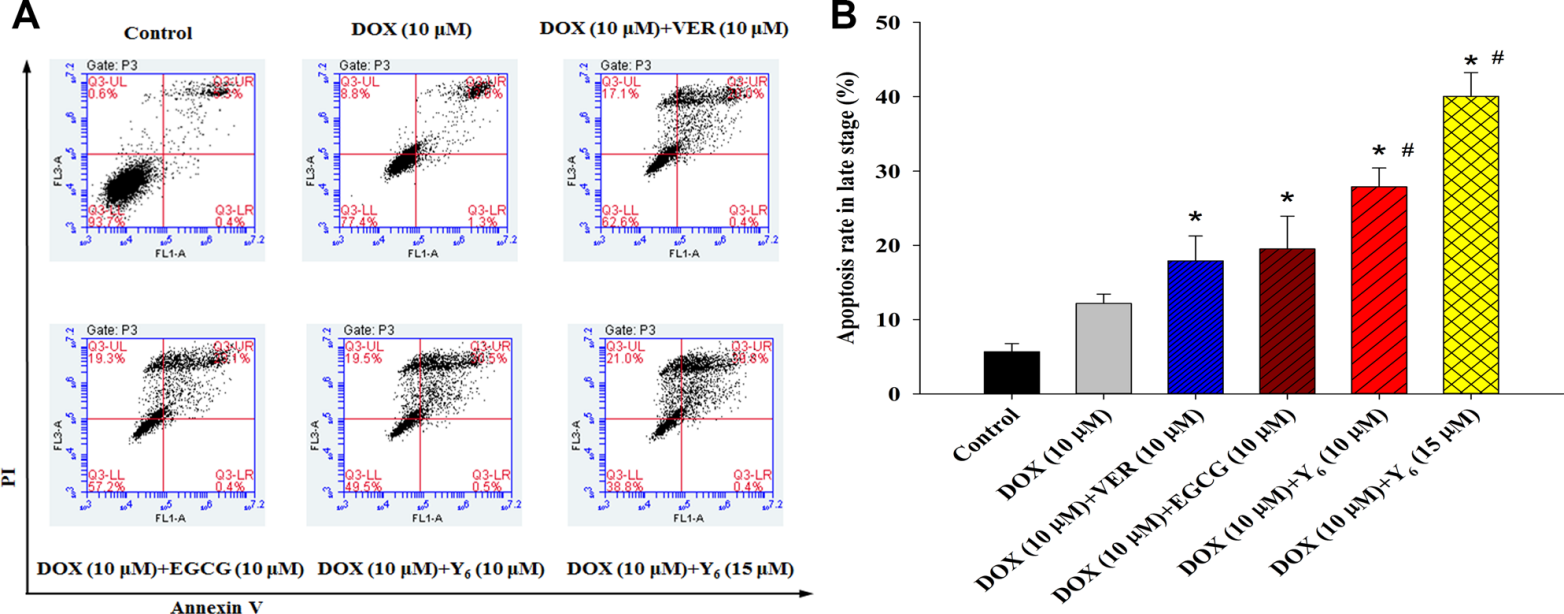
Apoptosis was measured in BEL-7404/DOX cell line (**A**) Apoptotic cells as a result of Y_6_, EGCG, and verapamil treatment were quantified by the Annexin V/PI assay. Cells were stained using an Annexin V/PI staining kit and then detected by flow cytometry. (**B**) *p < 0.05 vs. the doxorubicin alone group. ^#^*p* < 0.05 vs. the (verapamil 10 μM+doxorubicin 10 μM) group or (EGCG 1 0 μM+doxorubicin 10 μM) group. Experiments were performed for three independent times.

The induction of cell apoptosis is a common mechanism for many anti-tumor drugs. To examine whether Y_6_ can induce cell apoptosis, we detected apoptotic cells in HCC BEL-7404/DOX cells treated with verapamil (10 μM), EGCG (10 μM), and Y_6_ (10 and 15 μM) in combination with doxorubicin (10 μM) and compared with doxorubicin (10 μM) alone. The cells that treated with verapamil were served as the positive controls of P-gp inhibitors. BEL-7404/DOX cells were incubated in anoxic condition for 48 h, then subjected to Annexin V-FITC labeling and Propidium iodide (PI) staining as described in the Materials and Methods section. We used flow cytometry analysis to determine the apoptotic rate of BEL-7404/DOX cells treated with the drug combinations or doxorubicin alone. The results showed that after treatment with any drug combination, the number of cells increased in late apoptotic stage (Table 2, Figure 3). Only 12.17% of cells showed apoptotic signals when treated with doxorubicin alone, but the percentage increased to 17.91% with verapamil (10 μM), to 19.52% with EGCG (10 μM), to 27.89% with Y_6_ (10 μM) and to 40.03% withY_6_ (15 μM). The differences were statistically significant when compared with the doxorubicin group (*p < 0.05). Furthermore, at the same concentration, Y_6_ had a higher effect than verapamil and EGCG on the induction of apoptosis in the late apoptotic stage, and the differences were statistically significant (^#^*p* < 0.05). In addition, the higher the concentrations of Y_6_, the more cell apoptosis in the BEL-7404/DOX cells.

**Table 2:** The late stage apoptosis rates in BEL-7404/DOX cells (*n* = 3)

Drugs (μM)	Apoptosis rate (%) ± SD (μM)^a^
Control^b^	5.67 ± 1.10
DOX 10 μM^c^	12.17 ± 1.26
+VER 10 μM^d^	17.91 ± 3.35*
+EGCG 10 μM^d^	19.52 ± 4.41*
+Y_6_ 10 μM^d^	27.89 ± 2.53^*#^
+Y_6_ 15 μM^d^	40.03 ± 3.21^*#^

a: Values represent means ± SDs of at least three independent experiments performed in triplicate. b: Control group, no drugs were used; c: DOX group, 10 μM of DOX alone was used; d: 10 μM of DOX combined with 10 μM of VER, 10 μM of EGCG, 10 μM of Y6, or 15 μM of Y_6_ were used; (VER 10 μM+DOX 10 μM) group was used as the positive control; *p < 0.05 vs. the DOX alone group. ^#^*p* < 0.05 vs. the (VER 10 μM +DOX 10 μM) group or (EGCG 10 μM +DOX 10 μM) group.

### Y_6_ inhibited the expression of hypoxia-inducible factor-1α(*hif-1α*) and *MDR1* mRNA in BEL-7404/DOX cells

**Figure 4:**
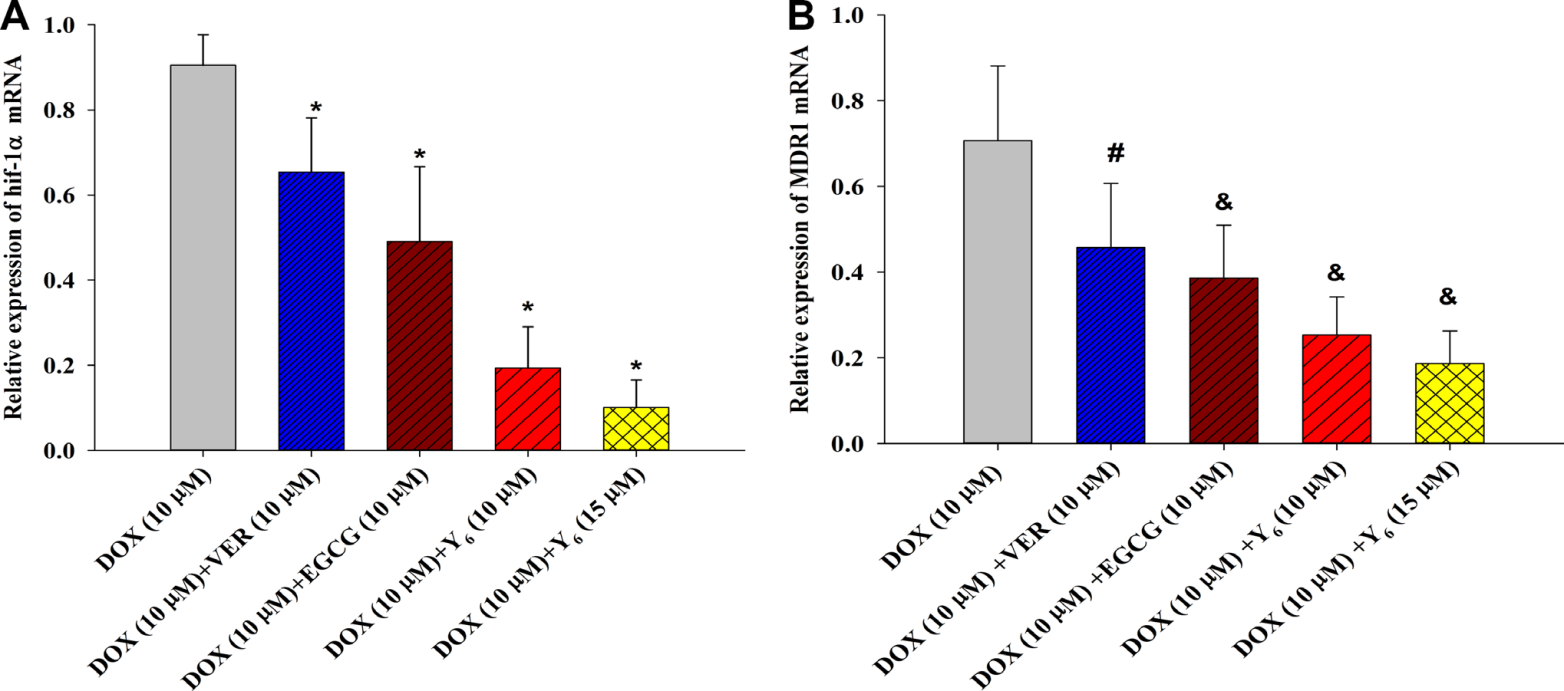
The expression of *hif-1α* and *MDR1* genes were measured in BEL-7404/DOX cells by RT-PCR analysis (**A**) *p < 0.05 vs. the doxorubicin alone group on the *hif-1α* gene; (**B**) ^#^*p* > 0.05 vs. the doxorubicin alone group on the *MDR1* gene; ^&^*p* < 0.05 vs. the doxorubicin alone group on the *MDR1* gene. Experiments were repeated three independent times.

Previous studies have shown that high *MDR1*-gene expression exists in BEL-7404/DOX cells compared with BEL-7404 cells [21, 22]. Therefore, differences in expressions of *hif-1α* and *MDR1* genes were investigated by quantitative real time polymerase chain reaction (RT-PCR) analysis after drug treatment. The results showed that the expression of *hif-1*α was significantly lower in the combination drug therapy groups (doxorubicin plus EGCG, verapamil, or Y_6_) than in the doxorubicin alone group. Expression of *hif-1*α was 0.90 in BEL-7404/DOX cells treated with doxorubicin (10 μM) alone, but it decreased to 0.65 with verapamil (10 μM), to 0.49 with EGCG (10 μM), to 0.19 with Y_6_ (10 μM), and to 0.10 with Y_6_ (15 μM). For the *MDR1* gene, the expressions were significantly lower in the combination drug therapy groups (doxorubicin plus EGCG or Y_6_, excluding verapamil) than that in the doxorubicin alone group. The expression of *MDR1* was shown to be 0.71 in BEL-7404/DOX cells treated with doxorubicin (10 μM) alone, but it decreased to 0.39 with EGCG (10 μM), to 0.25 with Y_6_ (10 μM), and to 0.19 withY_6_ (15 μM). The differences were statistically significant not only in *hif-1α*, but also in *MDR1*, when compared with the doxorubicin group (*p* < 0.05) (Table 3, Figure 4). These results indicated that treatment of Y_6_ decreased the expression of *hif-1α* and *MDR1* mRNA.

**Table 3:** The expression of hif-1α and MDR1 genes in BEL-7404/DOX cells (*n* = 3)

Gene Names	2^−ΔΔCT^ ± SD^a^					
	Control	DOX(10)^b^	EGCG(10) + DOX^c^	VER(10) + DOX^c^	Y6(10) + DOX^c^	Y6(15) + DOX^c^
*hif-1α*	——	0.90 ± 0.07	0.49 ± 0.18*	0.65 ± 0.13*	0.19 ± 0.10*	0.10 ± 0.06*
*MDR1*	——	0.71 ± 0.18	0.39 ± 0.12^&^	0.46 ± 0.15^#^	0.25 ± 0.09^&^	0.19 ± 0.08^&^

The expression of *hif-1α* and *MDR1* genes were measured in BEL-7404/DOX cell by RT-qPCR analysis. a: Values represent means ± SDs of at least three independent experiments performed in triplicate; b:DOX group, 10 μM of DOX alone was used; c:10 μM of DOX combined with 10 μM of VER, 10 μM of EGCG, 10 μM of Y_6_, or 15 μM of Y_6_ were used; VER (10)+DOX (10) was used as the positive control; Control group, no drugs were used; *p < 0.05 vs. the DOX alone group on the *hif-1α* gene; ^&^*p* < 0.05 vs. the DOX alone group on the MDR1 gene; ^#^*p* > 0.05 vs. the DOX alone group on the MDR1 gene.

### Y_6_ inhibited the expression of HIF-1α and P-gp proteins in BEL-7404/DOX cells

**Figure 5:**
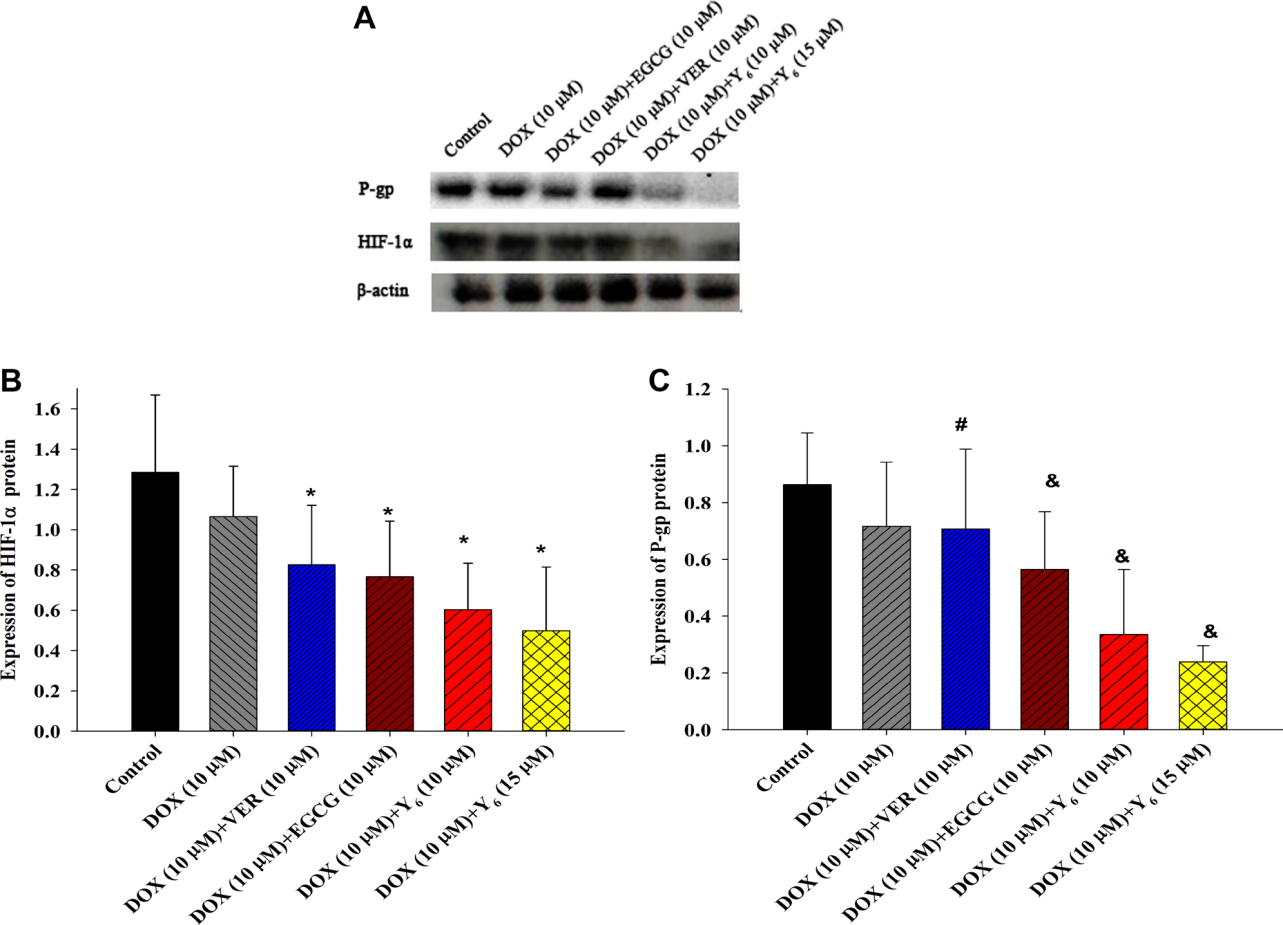
The expression of HIF-1α and P-gp proteins (**A**) The expression of HIF-1α and P-gp proteins were measured in BEL-7404/DOX cells by Western blotting analysis. (**B**) (**C**) Relative protein levels were quantified by densitometry and showed in the histogram. Values represent means ± SDs of at least three independent experiments performed in triplicate; *p < 0.05 vs. the doxorubicin alone group on the HIF-1α protein; ^#^ *p* > 0.05 vs. the doxorubicin alone group on the P-gp protein; ^&^*p* < 0.05 vs. the doxorubicin alone group on the P-gp protein. Experiments were repeated three independent times.

Since PCR results showed that treatment with Y_6_ inhibited the mRNA expressions of *hif-1α* and *MDR1* genes compared with the doxorubicin alone group, we investigated whether the expressions of their encoded proteins HIF-1α and P-gp were also inhibited. The expressions of HIF-1α and P-gp proteins were examined by Western blotting analysis. The results showed that the expressions of HIF-1α and P-gp proteins were significantly decreased in the combination drug therapy groups (doxorubicin plus Y_6_) compared to the doxorubicin alone therapy group (*p < 0.05 and ^&^*p* < 0.05) (Figure 5).

## DISCUSSION

Nature is an endless source of organic compounds for development of new drugs including resistance reversal agents for cancer. EGCG, from the Chinese green tea, is the effective ingredient for reversing anti-cancer drug resistance. Alkylate modification is one way to remedy the EGCG's defects of low lipid solubility and stability [23]. Previous studies have shown that the ethylated and propyl derivatives of EGCG had strong reversal effect, while EGCG-methylated derivatives had no effect on the MDR in hepatocellular carcinoma cells [24]. However, with the increase of carbon chain, the toxicity to normal hepatocyte is also increased. Therefore, the propyl derivatives were more appropriate to be the potential anticancer drugs [24]. We synthesized six different EGCG-ethylated derivatives. Among them, Y_6_ had not only more stable features but also better lipid solubility when compared with EGCG and other EGCG-ethylated derivatives. In this study, we elucidated the effect and mechanism of Y_6_ in reversing drug resistance in BEL-7404/DOX cells.

By combining non-cytotoxic concentrations of Y_6_ (below the IC_10_ values) with doxorubicin, we determined that Y_6_ potentiated the doxorubicin cytotoxic effects on cancer cells. Our MTT assay result showed that Y_6_ and EGCG could enhance the cytotoxicity of doxorubicin by reversing multidrug resistance in BEL-7404/DOX cells. In addition, flow cytometric analysis result revealed that Y_6_ increased cell apoptosis in the late apoptotic stage. The reversal effects of Y_6_ were higher than that of EGCG both in MTT assay and in flow cytometric analysis experiments. Our study demonstrated that Y_6_ has excellent ability in increasing cell sensitivity to doxorubicin and inducing apoptotic cell death.

Hypoxia is a common feature in solid tumors and is generally associated with tumor chemo-resistance [25, 26]. Hypoxia may reduce tumor sensitivity to radiation therapy and chemotherapy by deprivation of the oxygen essential for the cytotoxic actions directly, and lead to increasing resistance by inducing proteomic and genomic changes indirectly [27]. In the study by Du Cetal, hypoxia was shown to enhance tumor development in HCC [28]. In the experiment by Yu G et al, the chemosensitivity to 5-FU or cisplatin in HBx-HepG2 cells was measured by MTT assay in different hypoxic (5% O_2_, 3% O_2_, or 1% O_2_) conditions. The results revealed that under hypoxic conditions, particularly at 1% O_2_, the cancer killing effects of 5-FU or cisplatin were prominently abrogated [29]. Therefore our experiment adopted the hypoxic condition of 1% O_2_ to imitate the hypoxic environment of solid tumors *in vivo*. HIF-1 is a central component of hypoxic adaptation. It has been presented as an important mediator of hypoxia-regulated gene expression and can activate the transcription of genes that are important to cell viability, invasion, metastasis, and angiogenesis [30–32]. Hypoxia may significantly increase the protein expression level of HIF-1α [29]. The activation of HIF-1 is closely related to the increased of expression of *MDR1* gene and its product P-gp protein [33–38]. P-gp protein was the first transporter to be identified that associated to MDR. It was also the most representative and widely studied in reversing resistance. Previous study showed that expression of HIF-1α and MDR1/P-gp could be regarded as a predictive marker of chemotherapy resistance, and HIF-1α inhibition was use to reverse multidrug resistance by reducing expression of MDR1/P-gp [39]. Other studies revealed that the expressions of *hif* mRNA and its encoded protein were decreased after treatment with verapamil [40], but there was no decrease in the level of P-gp protein [41].

To investigate whether the effect of Y_6_ on the reversal of resistance was associated with decreased P-gp expression via the HIF-1α-mediated pathway, we measured the mRNA expressions of *hif-1α* and *MDR1* genes by quantitative RT-PCR analysis and expressions of their encoded proteins of HIF-1α and P-gp by Western blotting. The BEL-7404/DOX cells were pretreated in hypoxic condition before experiment. The results of our study revealed that expressions of *hif-1*α gene and its encoded protein were significantly down-regulated upon the treatment with doxorubicin plus Y_6_. We also observed a significant decreased expression of *MDR1* gene and its encoded protein P-gp in parallel with a decrease in HIF-1α expression in BEL-7404/DOX cells. The coincident decline of HIF-1α and MDR1/P-gp expression treated with Y_6_ suggested a positive correlation between the two proteins. Y_6_ were more potent in decreasing the expressions of HIF-1α and P-gp both in gene and in protein level than EGCG. The strong reversal effect of Y_6_ was most likely due to the decline of P-gp transporter.

In conclusion, the present investigation suggested that Y_6_ might reverse P-gp-mediated MDR. Y_6_ in combination with doxorubicin could increase the cytotoxicity of doxorubicin *in vitro*. The mechanisms of Y_6_ in reversing MDR are likely to be related to the HIF-1α-*MDR1*/P-gp pathway. Y_6_, as a novel inhibitor resulting from the modification of natural product, may become an effective drug to overcome MDR in the treatment of hepatocellular carcinoma.

## MATERIALS AND METHODS

### Materials

EGCG (purity 97%) from green tea was obtained from Hangzhou Hetian biotechnology Co., Ltd. (Hangzhou, Zhejiang, China). Y_6_ (purity 96.87%) was synthesized by our research group and was dissolved in dimethylsulfoxide (DMSO) to form a stock solution of 10 mM and stored at −20°C. Verapamil hydrochloride was obtained from Shanghai Hefeng Pharmaceutical Co., Ltd. (Shanghai, China). Doxorubicin was purchased from Shanxi Pude Pharmaceutical Co., Ltd. (Datong, Shanxi, China). DMSO was purchased from Guangzhou Xingang Co., Ltd. (Guangzhou, Guangdong, China). 3-(4, 5-dimethylthiazol-2-y1)-2, 5-diphenyltetrazolium bromide was purchased from Sigma (St. Louis, MO). An Annexin V-FITC/ Propidium iodide (PI) staining assay kit was obtained from Sony Biotechnology Inc. (San Jose, CA). Total Ribonucleic Acid (RNA) extraction reagent kit (RNAiso plus) and Prime Script^TM^ RT reagent Kit with cDNA Eraser for Perfect Real Time were obtained from TAKARA Bio Inc. (Dalian, China). The anti-HIF-1α, anti-P-gp antibody, and anti-rabbit IgG (H+L) (Dy Light 680 Conjugate) were purchased from Cell Signaling Technology (Beverly, Massachusetts). β-actin polyclonal antibody was obtained from Bioworld Technology, Inc. (St. Louis Park, MN). RPMI-1640 medium was obtained from Hyclone Laboratories (Logan, UT), and fetal bovine serum (FBS) was from Hangzhou Sijiqing Co., Ltd. (Hangzhou, Zhejiang). Penicillin-Streptomycin Liquid (PS; 10,000 U/mL) and trypsin-EDTA Solution (containing 0.25% trypsin and 0.02% EDTA) were obtained from Beijing Solarbio Science&Technology Co., Ltd. (Beijing, China).

### Hypoxia cell culture

Cells were cultured under normoxic condition (2% O_2_, 5% CO_2_, 74% N_2_) for 24 h and subsequently under hypoxic condition (1% O_2_, 5%CO_2_, 94% N_2_) for 48 h in the incubator (NU4950, NUAIRE company, USA) [29].

### Cell culture

The parental HCC cell line BEL-7404 and the drug resistant HCC cell line BEL-7404/DOX were obtained from the Department of Physiopathology, Guangxi Medical University (Nanning, Guangxi, China). The doxorubicin-resistant cell line BEL-7404/DOX had been developed by exposure of cells to doxorubicin at gradually increasing doses. BEL-7404 and BEL-7404/DOX cells were maintained in RPMI-1640 medium containing 10% (v/v) FBS and 1% (v/v) PS at 37°C in a normoxic incubator (Forma Scientific, Inc., Marietta, OH). BEL-7404/DOX cells were cultured with doxorubicin (0.1 μg/mL) to maintain drug resistance. For subculturing, cells were dissociated by using 0.25% trypsin.

### Cell viability assay

The cytotoxicity of drugs to HCC cells (BEL-7404 and BEL-7404/DOX) was measured by the MTT assay [39]. Cells were seeded in 96-well plates at a density of 5000 cells/well for 48 h. The cells were treated with various concentrations of Y_6_, EGCG, verapamil, and doxorubicin, or doxorubicin in combination with Y_6_, EGCG, and verapamil for 48 h in hypoxic condition. Then MTT (20 μL; 5 mg/mL) was added to each well for an additional 4 h. The medium was removed and 100 μL of DMSO was added to each well to dissolve the formazan crystals that had formed. The optical density at 490nm was measured on a multi-well plate reader (Bio-Rad, Hercules, CA). The percentage of cell survival was calculated using the background-corrected absorbance, as follows: % Cell viability = (OD_experimental_/OD_control_) × 100.

All experiments were repeated 3 times. IC_50_ values are drug concentrations at which there is 50% reduction in optical density. The fold of drug resistance was calculated as the IC_50_ of BEL-7404/DOX divided by the IC_50_ of BEL-7404. The degree of reversal of drug resistance (reversal fold or RF) was calculated as the IC_50_ with doxorubicin alone divided by the IC_50_ with doxorubicin in combination with other drugs (Y_6_, EGCG, and verapamil).

### Annexin-V-FITC/PI staining assay

The Bel-7404/DOX cells were incubated for 48 h, and then were treated with medium alone, medium with doxorubicin alone, doxorubicin+Y_6_, doxorubicin+EGCG, or doxorubicin+verapamil for 48 h. Subsequently, the cells were subjected to flow cytometry analysis. The Bel-7404/DOX cells were suspended in Annexin V Binding Buffer at a concentration 0.25~1.0 × 10^7^ cells/ml. Five μL of FITC and 10 μL of PI were added to 100μL of cell suspension per tube in turn. The cells were mixed in vortex and incubated for 15 minutes at room temperature (25°C) in the dark. Annexin V Binding Buffer (400 μL) was added to each tube. The mixed cell suspensions were analyzed by flow cytometry. The fluorescence of the sample was detected by Epics XL Flow Cytometry (Beckman Coulter) (Ex = 488 nm and Em = 525 nm for Annexin V-FITC; Ex = 488 nm and Em = 620 nm for PI).

### RT-PCR analysis

The BEL-7404/DOX cells were incubated in hypoxia (1% O_2_) for 48 h, and then were treated with medium alone, medium with doxorubicin alone, doxorubicin+Y_6_, doxorubicin+EGCG, or doxorubicin+verapamil for 48 h. Subsequently, the cells were subjected to RT-PCR analysis. Total mRNA of the BEL-7404/DOX cells was extracted using Trizol reagent according to manufacturer's instructions, and resuspended in RNase water. The content and purity of total RNA in suspension was measured by ultraviolet spectrophotometer (wave length 260 nm), and the integrity of RNA was checked with the Bioanalyser 2100. One μg of total RNA was reverse transcribed (RT) to cDNA by using the PrimeScript and RT Primer Mix (including Oligo dt Primer and Random 6 mers). For PCR amplification of cDNA, 2 μL of cDNA liquid was added to 23 μL of PCR reaction media (including SYBR Premix Ex Taq, PCR Forward/Reverse Primer, and double distilled water). PCRs were carried out in the conditions according to the instructions: initial denaturation (95°C for 30 s) followed by 40 cycles of denaturation (95°C for 5s), and hybridization-extension (60°C for 31s) using Applied Biosystems7300 Real Time PCR System. The gene names (including target genes *hif-1α* and *MDR1* and housekeeping gene ACTB), access number, primer sequence, and amplifying size are listed in Table 4. In the results, transcription levels of target genes were normalized to those of ACTB to compensate for difference in efficiency of reverse transcription and input RNA amounts; they were expressed relative to the control. The relative quantitation of target gene expression was calculated by the comparative ΔΔCT method.

**Table 4:** Primers used for the RT-qPCR studies

Gene	Genebank access number	Primer sequence (5′–3′)	Amplicon size (bp)
*hif-1α*	NM_001243084.1 [42]	Forward- GACACAGAAGCAAAGAACCCA Reverse- CATCAGTGGTGGCAGTGGTA	241
*MDR1*	NM_000927.4 [43]	Forward-CTCTTTGCCACAGGAAGCCT Reverse- CATTGCGGTCCCCTTCAAGA	187
*ACTB*	NM_001101.3 [44]	Forward-GGCATCCTCACCCTGAAGTA Reverse-GCACACGCAGCTCATTGTAG	102

### Western blot analysis

The BEL-7404/DOX cells were incubated in hypoxia (1% O_2_) for 48 h, and then were treated with medium alone, medium with doxorubicin alone, doxorubicin+Y_6_, doxorubicin+EGCG, or doxorubicin+verapamil for 48 h. Subsequently, the cells were subjected to Western blot analysis. Cells with different treatments were lysed in an ice-cold buffer containing 50 mM Tris (pH 7.4), 150 mM NaCl, 1% Triton X-100, 1% sodium deoxycholate, 0.1% SDS, sodium orthovanadate, sodium fluoride, EDTA, leupeptin, 1× protease inhibitor cocktail, and 1×phosphatase inhibitor cocktail at 4°C for 30 min. Cell lysates were centrifuged at 4°C with 13,000 rpm/min for 10 min. Supernatant of cell lysates was transferred to the Eppendorf tube and measured for protein concentrations.

Twenty-five micrograms of protein liquid from each sample were placed in electrophoresis tanks in a 10–12% SDS polyacrylamide gel. The cell lysates were separated on the SDS polyacrylamide gel and then transferred electrophoretically onto a nitrocellulose membrane, which was pre-hybridized in methyl alcohol. The membrane was submerged in 5% skim milk for 1 h, and then transferred to a solution containing 20 mM Tris-HCl (pH 7.4), 150 mM NaCl, 0.05% Tween-20 (TBST buffer) and a primary antibody and incubated overnight at 4°C. After washing with the TBST buffer, the membrane was submerged in TBST buffer containing a Dy Light 680 secondary antibody in the dark at room temperature for 2 h. The membrane was washed with TBST buffer and then exposed to Infrared Imaging System to visualize the bands.

### Statistical analysis

The results of each experiment were represented as the mean ± standard deviation (SD) and analyzed in SPSS 20.0 software. One-Way ANOVA method was used in multiple comparison tests. A difference was considered significant when *p value* was less than 0.05.
